# Dramatic Enhancement and Integration of Properties in Pork Belly‐Inspired All‐Biomass Composite Films via Facile, Sustainable, and Controllable Design

**DOI:** 10.1002/advs.78388

**Published:** 2026-10-22

**Authors:** Junchao Ren, Chenglei Huang, Chengxu Pei, Mengde Huang, Yuanyuan Xu, Chengyu Yu, Hao Nie, Guangbing Zhao, Shijing Song, Hanwu Lei, Fang Wang, Bin Li, Qingfa Zhang

**Affiliations:** ^1^ School of Mechanical and Vehicle Engineering School of Intelligent Manufacturing Anhui Agricultural University Hefei China; ^2^ Department of Biological Systems Engineering Washington State University Richland USA; ^3^ College of Agricultural Engineering and Food Science Shandong University of Technology Zibo China

**Keywords:** all‐biomass, alternating multilayer, electromagnetic interference shielding, thermal management

## Abstract

Although all‐biomass composite films that integrate electromagnetic interference (EMI) shielding, thermal management, and mechanical properties are promising alternatives to conventional flexible electronic materials owing to the renewability, degradability, and sustainability, the substantial performance shortcomings severely limit the practical availability of these materials. In this study, an effective layer‐by‐layer assembly strategy inspired by the hierarchical structure of pork belly was proposed to fabricate alternating multilayer (AM) biomimetic composite films composed of cellulose nanofibers (CNFs), graphitized carbon nanosheets (GCNs), and gelatin. Efficient stress transfer, phonon transport, and electromagnetic attenuation were achieved through the construction of a robust hierarchical multilayer architecture enabled by the synergistic interactions among GCNs, CNFs, and gelatin. The results showed that the AM‐7 composite film exhibited a tensile strength of 121.20 MPa, which was increased by 266% and 199% compared with CF and CG composite films, respectively. An enhanced in‐plane thermal conductivity of 6.34 W·m^−^^1^·K^−^^1^ was achieved, and the EMI shielding effectiveness attained 56.35 dB. This work provides a sustainable strategy for the high‐value utilization of agricultural waste while offering new insights into the rational design of high‐performance multifunctional biomass‐based composites.

## Graphical abstract

A pork belly‐inspired all‐biomass composite film was constructed through layer‐by‐layer assembly of cellulose nanofibers (CNFs), gelatin, and graphitized carbon nanosheets (GCNs) via hydrogen bonding and π–π stacking interactions. The excellent mechanical properties, effective thermal management, and outstanding EMI shielding performance of the alternating multilayer composite film provide a sustainable strategy for multifunctional flexible electronic applications.

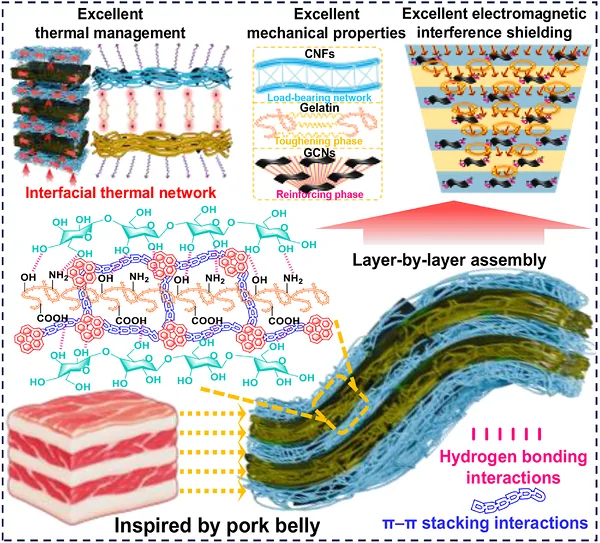

## 1 Introduction

Electromagnetic interference (EMI) shielding materials have attracted considerable attention due to their important applications in electronic communications, flexible electronic devices, aerospace, and wearable devices [1, 2]. The primary function is to attenuate electromagnetic wave propagation through reflection, absorption, and multiple scattering mechanisms to ensure the stable operation of electronic systems and information security [3, 4]. Recently, EMI shielding materials can be generally classified into metal‐based materials (e.g., Cu, Al, Ni) [5], carbon‐based materials (e.g., carbon nanotubes, graphene, carbon fibers) [6], conductive polymers (e.g., polyaniline, polypyrrole) [7], and emerging 2D materials (e.g., MXene, MoS_2_) [8]. Considerable progress has been achieved in these materials for EMI shielding applications. Despite this, inherent trade‐offs between electrical conductivity and flexibility, insufficient structural stability, and difficulties in multifunctional integration are major challenges in complex practical environments [9, 10]. Meanwhile, most raw materials with high cost, limited scalability and poor environmental stability fail to meet the requirements of smart home devices and flexible electronic equipment [11, 12]. Therefore, it is highly desirable to develop lightweight, sustainable, and cost‐effective EMI shielding materials derived from renewable resources.

Amid increasing global resource scarcity and environmental challenges, the high‐value utilization of renewable biomass resources has emerged as a key research focus in materials science [13, 14]. Lignocellulosic biomass is considered an important candidate for replacing conventional petrochemical resources due to wide availability, renewability, biodegradability, and carbon neutrality [15]. In particular, large amounts of straw‐based agricultural waste generated during agricultural production are rich in natural components such as cellulose, hemicellulose, and lignin, providing abundant raw material sources for the development of high‐performance functional materials [16]. It cannot be ignored that most agricultural wastes are still mainly disposed of through incineration, landfilling, or low‐value utilization, which not only results in resource waste but also causes environmental pollution [17]. Thus, there is an urgent need to develop high‐value functional materials from agricultural waste to achieve the synergistic integration of resource recycling and performance enhancement. In this context, biomass‐derived functional composites have been extensively developed as an effective strategy to upgrade low‐value biomass into high‐performance materials [18]. Among them, all‐biomass composites have attracted considerable attention due to renewable nature, biodegradability, and environmental friendliness, and have emerged as a promising class of sustainable functional materials [19, 20]. Materials constructed from natural biopolymer components such as cellulose, proteins, and polysaccharides via multiscale structural regulation strategies including nanocrystal templating, self‐assembly, freeze‐drying, and 3D printing have demonstrated significant advantages in functional integration [21]. Previous studies have shown that all‐biomass systems have been widely explored across diverse fields, including information functional materials (structural color and circularly polarized luminescence for information encryption and anti‐counterfeiting) [22]; flexible electronic devices (highly sensitive strain and pressure sensors for human motion monitoring) [23]; energy and environmental applications (photothermal evaporation for seawater desalination and wastewater treatment, and radiative cooling for passive thermal regulation and energy management) [24]; as well as functional structural materials (aerogels for thermal insulation and flame‐retardant applications) [25]. These advances collectively demonstrate that all‐biomass materials not only enable multifunctional integration, but also show great promise as sustainable alternatives to conventional non‐degradable materials. In addition, our previous studies have developed several all‐biomass‐derived multifunctional composite film systems with integrated thermal management, EMI shielding, and mechanical performance. For instance, self‐assembled nano carbon spheres (NCSs) and cellulose nanofibers (CNFs) achieved a tensile strength of 41.74 MPa, an in‐plane thermal conductivity of 2.21 W m^−^^1^ K^−^^1^, and an EMI shielding effectiveness of 23.77 dB [26]. Also, a hydrogen‐bonded organic framework–carbon nanosheets/CNF (HOFs–CNs/CNF) composite film was constructed via in situ growth and self‐assembly, exhibiting improved multifunctional integration, including a tensile strength of 50.58 MPa, an in‐plane thermal conductivity of 4.57 W m^−^^1^ K^−^^1^, and an EMI shielding effectiveness of 32.11 dB [27]. Furthermore, an all‐biomass‐derived carbon quantum dots/CNs/CNFs system was designed, delivering a thermal conductivity of 0.817 W m^−^^1^ K^−^^1^, a tensile strength of 39.60 MPa, and excellent biodegradation behavior with residual rates of 86.02% (water), 66.67% (PBS), and 52% (burial conditions) [28]. However, achieving high‐performance biomass‐based systems with integrated mechanical, thermal, and EMI shielding performance to meet the requirements of advanced applications remains a significant challenge.

Based on this, an effective layer‐by‐layer assembly strategy inspired by the hierarchical structure of pork belly was proposed for constructing biomimetic biomass‐based composite films. The effects of multilayer structures on microstructure, mechanical properties, thermal conductivity, and EMI shielding performance are systematically investigated by tuning the number of layers and interfacial density, and the underlying synergistic interfacial reinforcement mechanism is further elucidated. This study provides a new strategy for the high‐value utilization of agricultural waste and offers guidance for the design of high‐performance multifunctional biomass‐based composites.

## 2 Experimental Section

### 2.1 Materials

Wheat straw powder was collected from a local farm in Anqing, Anhui Province, China. Potassium hydroxide (KOH), choline chloride (ChCl), glacial acetic acid (CH_3_COOH), sodium chlorite (NaClO_2_), and oxalic acid dihydrate (C_2_H_2_O_4_·2H_2_O) were purchased from Aladdin Reagent Co., Ltd. (Shanghai, China). Hydrochloric acid (HCl, 36–38 wt.%) was obtained from Chengdu Jinshan Chemical Reagent Co., Ltd. (Chengdu, China). Gelatin was obtained from Sinopharm Chemical Reagent Co., Ltd. (Shanghai, China). Ultrapure water (resistivity ≥ 18.2 MΩ·cm) was used throughout all experiments.

### 2.2 Preparation of Graphitized Carbon Nanosheets (GCNs) Through Sequential Graphitization and Flash Joule Heating of Wheat Straw‐Derived Carbon

Graphitized carbon nanosheets (GCNs) were prepared from wheat straw through carbonization, chemical activation, high‐temperature graphitization, and subsequent flash Joule heating treatment. Briefly, the wheat straw precursor was first pre‐carbonized at 500°C for 2 h under a nitrogen atmosphere with a heating rate of 10°C min^−^^1^. The obtained product was subsequently acid‐washed with hydrochloric acid to remove residual inorganic species and rinsed with ultrapure water until neutral. The purified carbon was then impregnated with 2 M KOH solution at a carbon‐to‐KOH mass ratio of 1:3 and dried at 80°C. The dried mixture was graphitized at 1600°C for 2 h under a nitrogen atmosphere. Subsequently, the graphitized precursor was subjected to flash Joule heating (FJH) under 160 V and 3 Ω to achieve an ultrahigh transient temperature.

### 2.3 Preparation of Cellulose Nanofibers (CNFs)

Cellulose nanofibers (CNFs) were prepared according to our previously reported method [27]. Briefly, wheat straw powder was delignified using 0.1 M sodium chlorite solution (pH 4–5) at 75°C for 1 h, and the treatment was repeated five times. The obtained material was subsequently treated with 2 wt.% KOH solution at 90°C for 2 h to remove residual lignin and hemicellulose. The purified cellulose was then treated with a deep eutectic solvent composed of choline chloride and oxalic acid dihydrate (1:1 molar ratio) at 110°C for 1 h. Finally, the cellulose suspension (2 mg/mL) was ultrasonicated at 900 W for 30 min to obtain CNFs.

### 2.4 Preparation of Alternating Multilayer Composite Films

CNFs/GCNs (CG) composite films were prepared by vacuum filtration and drying of a homogeneous suspension containing CNFs and GCNs at a mass ratio of 1:1. The resulting suspension was prepared at a concentration of 4 mg·mL^−^^1^, and 50 mL of the suspension was uniformly filtered using a glass frit filtration device to prepare a CG layer. After vacuum filtration, the resulting wet film was placed between two glass plates and dried at 60°C for 24 h in a conventional air oven. The thickness of the dried CG composite films was approximately 0.18 mm. Gelatin/GCNs (GG) composite films were fabricated by dissolving 2 g gelatin in 10 mL ultrapure water at 60°C to obtain a homogeneous gelatin solution, followed by the addition of GCNs at a gelatin‐to‐GCNs mass ratio of 1:1. The resulting homogeneous suspension was subjected to vacuum degassing and then uniformly cast onto a polytetrafluoroethylene (PTFE) plate. The cast suspension was dried at 60°C for 4 h. The thickness of the resulting GG was approximately 0.13 mm. Alternating multilayer composite films were assembled using CG composite films and GG composite films as the constituent layers. The composite films were stacked alternately and compressed under a pressure of 20 MPa for 30 min to improve interfacial adhesion. By varying the number of stacked layers, alternating multilayer composite films with 3, 5, and 7 layers were obtained and denoted as AM‐3, AM‐5, and AM‐7 composite films, respectively. For comparison, CF represents pristine CNFs films.

### 2.5 Characterization

The morphology and microstructure of the AM‐7 was observed using a field‐emission scanning electron microscope (SEM, Sigma 500, ZEISS, Germany). The chemical functional groups of CNFs, GCNs, gelatin, and AM‐7 were analyzed using a Fourier transform infrared (FTIR) spectrometer (Nicolet iS50, Thermo Fisher Scientific, USA) in the wavenumber range of 500–4000 cm^−^^1^. The crystal structures of CNFs, GCNs, gelatin, and AM‐7 were characterized using an X‐ray diffractometer (SmartLab SE, Rigaku, Japan) with a scanning range of 5–90° and a scanning rate of 5°/min. A thermogravimetric analyzer (TGA, TG 209 F1, NETZSCH, Germany) was used to evaluate the thermal stability of CNFs, GCNs, gelatin, and AM‐7 under a nitrogen atmosphere from room temperature to 900°C at a heating rate of 10°C min^−^^1^. Small‐angle X‐ray scattering (SAXS) and wide‐angle X‐ray scattering (WAXS) measurements were performed using a SAXS/WAXS system (Xeuss 2.0, Xenocs, France) to investigate the hierarchical structure and molecular ordering of the AM‐7 composite film. The elemental composition and chemical bonding states of the AM‐7 composite film were analyzed using an X‐ray photoelectron spectrometer (ESCALAB 250Xi, Thermo Fisher Scientific, USA). Density functional theory (DFT) calculations were performed using the DMol^3^ module in Materials Studio. The exchange–correlation interactions were described by the GGA‐PBE functional. A double numerical plus polarization (DNP) basis set was employed, and all structures were fully optimized before energy calculations. The convergence tolerances for energy, force, and displacement were set to 1 × 10^−^^5^ Ha, 0.002 Ha Å^−^^1^, and 0.005 Å, respectively.

### 2.6 Mechanical Properties of Alternating Multilayer Composite Films

A universal testing machine (UTM4104X, Sunstest Co., Ltd., China) was used to evaluate the mechanical properties of the composite films. The composite films were cut into standard dumbbell‐shaped specimens and tested at a crosshead speed of 10 mm/min, and the results were averaged from five measurements. Materials Studio 2023 was used to construct amorphous cells using the Amorphous Cell module. Molecular dynamics simulations were then performed with the Forcite module. The COMPASS III force field was applied to describe atomic interactions. Geometry optimization was first conducted to minimize the system energy. The systems were subsequently equilibrated under NVT and NVE ensembles. Finally, snapshots extracted at different simulation times were used to analyze the evolution of interfacial interactions between CNFs and gelatin.

### 2.7 Thermal Management Performance of Alternating Multilayer Composite Films

The in‐plane and out‐of‐plane thermal diffusivities of the composite films were measured using a laser flash analyzer (LFA 447, NETZSCH, Germany). The thermal conductivities were subsequently calculated according to λ = α·ρ·Cp, where α, ρ, and Cp represent the thermal diffusivity, density, and specific heat capacity, respectively. Finite element simulations were performed using COMSOL Multiphysics 6.0 to evaluate the thermal transport behavior of the composite films. A rectangular cuboid model with identical dimensions was constructed for all samples. The side surfaces were defined as thermally insulated boundaries. A constant temperature of 400 K was applied to the top surface, while the bottom surface was subjected to an identical heat flux load. The thermal conductivity of each sample was assigned according to the corresponding material properties, and all other simulation conditions were kept unchanged. The steady‐state temperature distributions were subsequently obtained and visualized using a unified temperature scale for comparison.

### 2.8 Electromagnetic Interference Shielding Performance of Alternating Multilayer Composite Films

The electromagnetic interference (EMI) shielding performance was evaluated in the X‐band frequency range (8.2–12.4 GHz) using a vector network analyzer (N5234A PNA‐L, Keysight Technologies, USA). Test samples were prepared using the rectangular waveguide method and carefully cut into strips measuring 22.9 mm × 10.2 mm to fit the specific waveguide sample holder.

R+A+T=1

$$R={\left|S_{11}\right|}^{2}={\left|S_{22}\right|}^{2}$$

$$T={\left|S_{12}\right|}^{2}={\left|S_{21}\right|}^{2}$$

where S11 and S21 denote the reflection and transmission coefficients, respectively. The total EMI shielding effectiveness (SE_T_) is composed of reflection loss (SE_R_), absorption loss (SE_A_), and multiple reflection loss (SE_MR_). SER originates from the impedance mismatch between the incident electromagnetic waves and the material surface, while SE_A_ is mainly associated with conductive loss, dielectric polarization, and interfacial polarization within the material. When SE_T_ is greater than 15 dB, the effect of multiple reflections can be neglected. Therefore, the shielding effectiveness contributed by reflection and absorption can be expressed as follows:

$$SE_{R}=10\;\log\left(\frac{1}{1-R}\right)=10\log\left(\frac{1}{1-{\left|S_{11}\right|}^{2}}\right)$$

$$SE_{A}=10\;\log\left(\frac{1-R}{T}\right)=10\log\left(\frac{1-{\left|S_{11}\right|}^{2}}{{\left|S_{21}\right|}^{2}}\right)$$

$$SE_{T}=SE_{R}+SE_{A}$$

The electromagnetic shielding efficiency (%) was calculated according to the following equation:

$$Shielding\;efficiency\;\left(\%\right)=100-\left(\frac{1}{10^{\frac{SE}{10}}}\right)\times 100$$

## 3 Results and Discussion

### 3.1 Morphology and Structural Characterization

Figure 1 illustrates the fabrication process and structural design of the AM biomimetic composite films. Inspired by the hierarchical architecture of pork belly, a biomimetic layer‐by‐layer assembly strategy was developed to prepare alternating multilayer structures using CNFs, GCNs, and gelatin. As shown in Figure 1A, CNFs were extracted from wheat straw through delignification, DES treatment, and ultrasonication, resulting in a highly entangled nanofibrous network rich in hydroxyl groups. Meanwhile, GCNs were prepared from wheat‐straw‐derived biochar through KOH activation, sequential graphitization, and flash Joule heating processes (Figure 1B), providing graphitized nanosheets with high electrical and thermal conductivity. Subsequently, CG composite layers and GG layers were alternately stacked through a layer‐by‐layer assembly strategy inspired by pork belly (Figure 1C). After hot pressing at 20 MPa for 30 min, a compact AM composite film with a well‐defined alternating multilayer architecture was obtained (Figure 1D). In addition, abundant hydrogen bonding between CNFs and gelatin, along with π–π stacking interactions among graphitized carbon nanosheets, contributed to the formation of a robust interfacial network.

**FIGURE 1:**
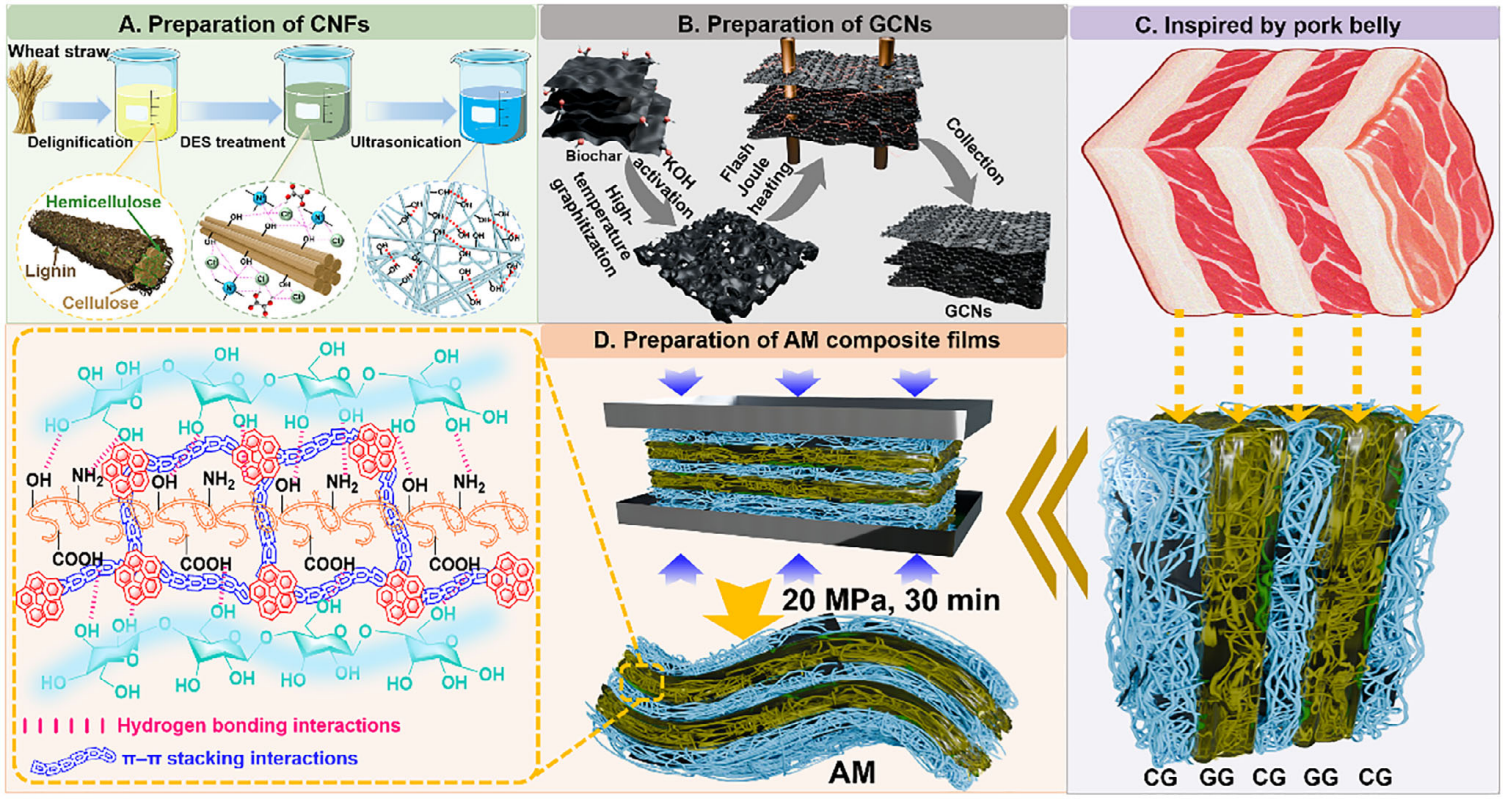
Schematic illustration of the preparation and biomimetic structural design of AM composite films. (A) Preparation of CNFs. (B) Preparation of GCNs. (C) Layered architecture of pork belly as the biological inspiration. (D) Preparation of AM composite films through layer‐by‐layer assembly and hot pressing.

As shown in Figure 2A, the AM‐7 composite film exhibits a well‐defined alternating multilayer architecture with closely stacked layers and distinct interfacial boundaries. The corresponding EDS elemental mapping (Figure 2B) reveals the uniform distribution of C and O throughout the fracture surface, while N is predominantly concentrated in specific layers, indicating the presence of gelatin‐rich regions and confirming the successful fabrication of the alternating multilayer architecture. The FTIR spectra of CNFs, GCNs, gelatin, and AM‐7 are shown in Figure 2C. A broad absorption band at approximately 3331 cm^−^^1^ is assigned to the stretching vibration of O–H groups. The absorption bands at 2900 and 1022 cm^−^^1^ are attributed to the stretching vibration of –CH_2_– groups and the C–O–C/C–O vibrations of the cellulose backbone, respectively [29]. In contrast, no obvious characteristic absorption peaks can be observed in GCNs due to the highly graphitized carbon structure and the limited amount of surface oxygen‐containing groups. Gelatin exhibits characteristic amide I, amide II, and amide III bands at 1650, 1540, and 1240 cm^−^^1^, respectively, which are associated with the peptide backbone of gelatin [30]. The characteristic peaks of CNFs and gelatin are retained in the AM‐7 composite film, while slight changes in peak intensity and position are observed, which can be attributed to the formation of intermolecular hydrogen‐bonding interactions between CNFs and gelatin [31]. Also, no new absorption peaks are observed in the AM‐7 composite film. This indicates that the composite was mainly formed through physical interactions rather than the formation of new covalent bonds. Figure 2D shows the XRD patterns of CNFs, GCNs, gelatin, and the AM‐7 composite film. Two characteristic diffraction peaks corresponding to the (110) and (200) crystal planes of cellulose I are observed for CNFs, indicating its semi‐crystalline structure. GCNs show a weak diffraction peak at around 26° corresponding to the (002) plane of graphitized carbon [32]. Also, several additional diffraction peaks appear at higher angles (e.g., 35.6°, 60.0°, and 71.8°), which can be assigned to reflections from graphitic domains. The observed diffraction features suggest a significantly enhanced graphitization degree resulting from the combined effects of high‐temperature carbonization and subsequent Joule heating, which promotes structural rearrangement and ordering of the carbon nanosheets. A broad diffraction peak can be observed in gelatin due to its amorphous nature. The XRD pattern of the AM‐7 composite film exhibits a broad diffraction feature in the 15–28° region, resulting from the overlap of crystalline CNFs and amorphous gelatin. Meanwhile, the sharper and more intense (002) diffraction peak of GCNs at ∼26° indicates enhanced graphitic ordering. No new diffraction peaks are observed, which suggests that the multilayer composite was formed through physical assembly without generating new crystalline phases. The Raman spectra before and after FJH show the characteristic D and G bands, confirming the structural transformation induced by FJH (Figure S1). Figure 2E,F shows the thermal stability of CNFs, GCNs, gelatin, and the AM‐7 composite film measured by TGA. Negligible weight loss over the entire temperature range is observed for GCNs due to its highly graphitized structure. CNFs and gelatin show significant mass loss in the range of 200–400°C corresponding to the decomposition of cellulose and protein‐based components, respectively. It should be noted that the AM‐7 composite film exhibits delayed degradation behavior (Figure 2F) and higher residual weight compared with the individual components. This suggests enhanced thermal stability of the multilayer structure. The presence of C, O, and N elements in XPS survey spectrum of the AM‐7 composite film shown in Figure 2G reveals successful integration of CNFs, gelatin, and GCNs. The high‐resolution C1s spectrum (Figure 2H) can be deconvoluted into three peaks at 284.8, 286.4, and 288.0 eV corresponding to C–C/C = C, C–O/C–N, and O–C = O/amide groups, respectively. The N1s spectrum (Figure 2I) exhibits a peak at around 400.0 eV, which is attributed to amine/amide nitrogen from gelatin. The O1s spectrum (Figure 2J) shows a peak at 532.6 eV corresponding to C─O and C═O bonds. These results further confirm the existence of abundant oxygen‐ and nitrogen‐containing functional groups, which facilitate strong interfacial interactions and hydrogen bonding within the AM‐7 composite film. The fracture surface SAXS/WAXS analysis of the AM‐7 composite film is shown in Figure 2K,L. A pronounced isotropic diffraction ring observed in the 2D WAXS pattern of the AM‐7 composite film suggests the presence of short‐range ordered or weakly stacked layered structures within the composite network. The non‐uniform intensity distribution along the ring suggests the existence of structural heterogeneity among the stacked domains, which is consistent with the designed multilayer architecture. Meanwhile, the 1D scattering profile further reveals two characteristic peaks at q = 1.13 Å^−^^1^ and 1.59 Å^−^^1^, which correspond to periodic distances of 0.556 and 0.395 nm, respectively. These results indicate the coexistence of multiple nanoscale packing domains arising from the synergistic assembly of CNFs, gelatin, and GCNs within the composite network. The corresponding 2D SAXS pattern (Figure 2L) displays a nearly isotropic scattering ring without obvious azimuthal anisotropy or streaks. This indicates randomly oriented nanoscale domains within the film. Although local ordered packing exists at the nanometer scale, the AM‐7 composite film shows no preferential orientation or long‐range directional alignment. In contrast, it forms an isotropic hierarchical network in which short‐range ordered domains are uniformly distributed throughout the composite matrix. The interaction mechanism within the AM composite system investigated by theoretical calculations is shown in Figure 2M. The optimized structures and charge distribution analysis reveal strong interfacial interactions among CNFs, gelatin, and GCNs, including hydrogen bonding, π–π stacking, and polar interactions [33]. The calculated total energy comparison demonstrates that all binary systems exhibit relatively higher energy states compared to the ternary AM‐7 system, which presents the lowest total energy [34]. The energy differences between AM‐7 and the binary systems further confirm that the introduction of the third component significantly stabilizes the interfacial interactions, leading to enhanced structural integrity of the composite network.

**FIGURE 2:**
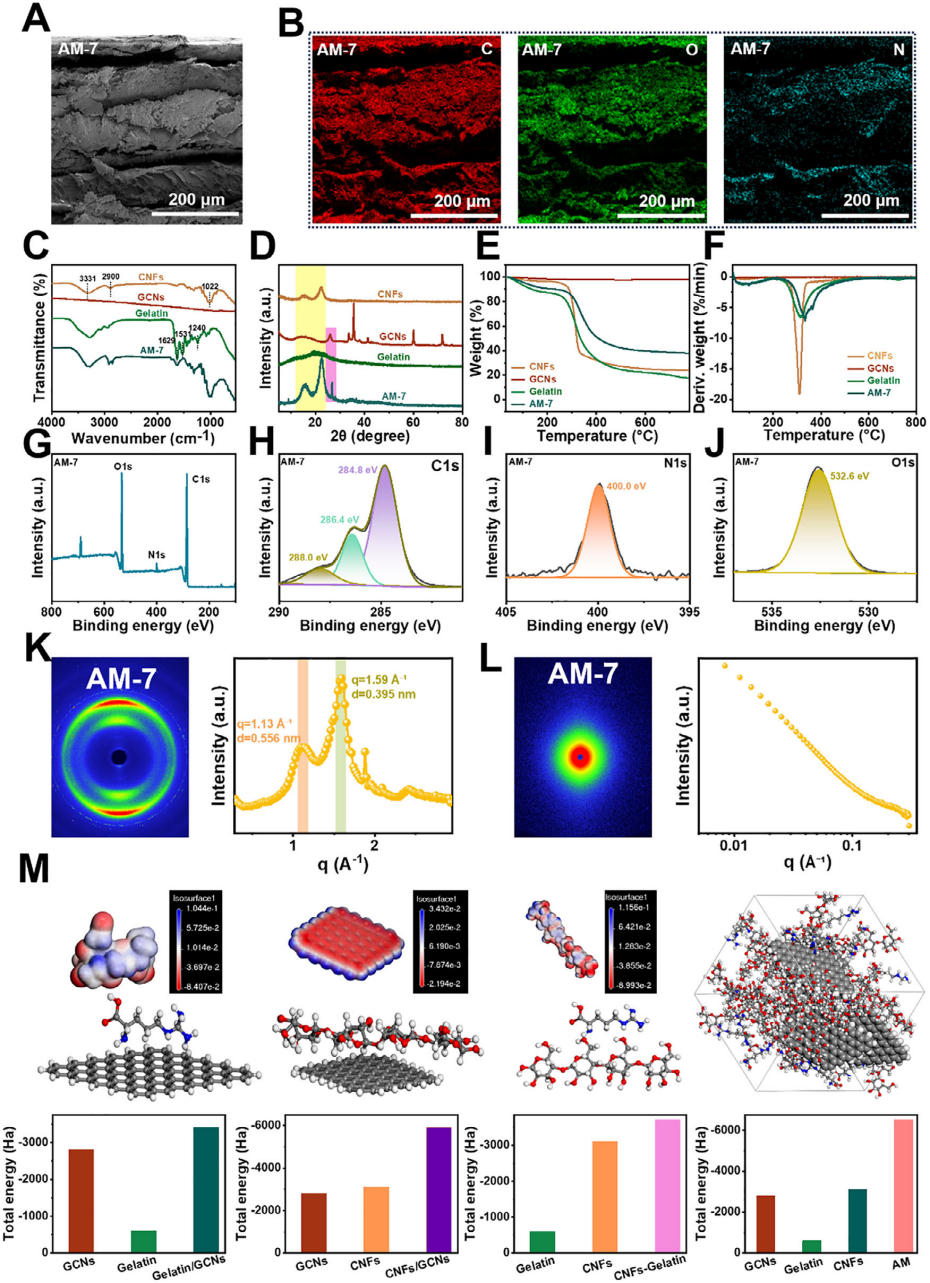
(A) SEM image of fracture surface and (B) elemental mapping of C, O, and N of the AM‐7 composite film. (C) FTIR spectra, (D) XRD patterns, (E) TG curves, and (F) DTG curves of CNFs, GCNs, gelatin, and the AM‐7 composite film. (G) XPS survey spectrum of AM‐7 composite film. High‐resolution XPS spectra of (H) C1s, (I) N1s, and (J) O1s. (K) 2D WAXS pattern and corresponding intensity profile, (L) 2D SAXS pattern and corresponding intensity profile of the AM‐7 composite film. (M) Theoretical calculations including electrostatic potential distribution and total energy analysis of CNFs, GCNs, gelatin, and AM‐7 composite film.

### 3.2 Mechanical Properties

Figure 3A shows the stress–strain curves of CF, CG, AM‐3, AM‐5, and AM‐7 composite films. The low strength and limited elongation of CF and CG indicate brittle fracture behavior. In contrast, all AM‐based composites show significantly enhanced mechanical properties. The tensile strengths of CF, CG, AM‐3, AM‐5, and AM‐7 composite films are 33.09, 40.52, 75.17, 100.30, and 121.20 MPa, respectively (Figure 3B). A progressive enhancement in tensile strength is observed with increasing layer‐by‐layer assembly and improved interfacial adhesion induced by hot pressing. It should be noted that the AM‐7 composite film exhibits a 266% increase compared with CF and a 199% increase compared with CG. This significant improvement is mainly attributed to the well‐designed multilayer architecture, which promotes more efficient stress transfer and suppresses crack propagation through a synergistic reinforcing effect between the layered components. Correspondingly, as shown in Figure 3C, the tensile moduli of CF, CG, AM‐3, AM‐5, and AM‐7 composite films are 1.35, 1.41, 1.46, 1.65, and 4.62 GPa, respectively. A pronounced increase in tensile modulus is observed for AM‐7 suggesting enhanced structural rigidity arising from the multilayer architecture and improved interfacial confinement after hot pressing. However, Figure 3D shows that the elongation at break values of CF, CG, AM‐3, AM‐5, and AM‐7 composite films are 3.27%, 4.38%, 7.96%, 5.58%, and 5.06%, respectively, showing an initial increase followed by a decrease. This trend is attributed to the balance between network deformability and structural rigidity due to the fact that improved interfacial interactions enhance deformation at lower stacking levels, while excessive structural densification in AM‐7 composite film restricts chain mobility. Figure 3E presents the toughness values of CF, CG, AM‐3, AM‐5, and AM‐7 composite films are 0.56, 0.97, 3.44, 3.08, and 3.60 MJ/m^3^, respectively. This indicates a significant enhancement in energy dissipation capability after structural assembly. Figure 3F,G compare the tensile strength and elongation at break of the AM‐7 composite film with previously reported all‐biomass composites, including carboxymethyl cellulose (CMC) [35], straw/WP (SW) [36], soy protein/starch/phytic acid (SSP) [37], mango kernel starch/guar/xanthan gums (MKS) [38], HOF‐based CNFs/CNF (SHCF) [27], and NCSs/CNFs (NCF) [26], as well as hybrid conductive composites such as ZIF‐8@ZIF‐67/MXene/TOCNF (ZMT) [39], BC@Fe_3_O_4_/CNT/Ti_3_C_2_Tx‐2L (BCTL) [40], CZIF/MXene/TOCNF (CMT) [41], CoNi@C/CNF/AgNWs (CCA) [42], PANI/AgNW (PA) [43], and CoFe_2_O_4_@MXene‐AgNW/CNF (CMAC) [44]. This demonstrates that the AM‐7 composite film achieves much higher mechanical strength while still maintaining a favorable balance between strength and ductility compared with most reported composites. As shown in the mechanical loading test (Figure 3H), the AM‐7 composite film can stably sustain increasing loads from 50 to 300 g without failure, which reflects its excellent load‐bearing capacity and structural integrity under external stress. Figure 3I illustrates the fracture evolution process of the gelatin–CNFs interface under tensile deformation. The simulated system consists of gelatin and cellulose chains, which represent the main interacting components in the composite film. The initially compact hydrogen‐bonded network gradually loosens with increasing strain, accompanied by the progressive rupture of interfacial interactions and the propagation of microcracks. Microcracks initiate at localized weak regions and progressively propagate along the interfaces, while the layered structure effectively deflects and hinders crack propagation [34]. In addition, the strong interfacial bonding and chain entanglement effectively delay crack propagation and contribute to energy dissipation during deformation. Also, the strengthening mechanism of the AM composite film schematically illustrated in Figure 3J. On the one hand, a continuous load‐bearing network was formed by CNFs as the primary skeleton for stress transfer. Gelatin was used as a toughening and binding phase, which formed hydrogen bonds with CNFs to enhance interfacial adhesion and facilitate energy dissipation during deformation. The improvement in mechanical strength was also attributed to the physical entanglement and mechanical interlocking effects induced by GCNs within the AM nanostructure [45]. On the other hand, the dissipation of stress, suppression of crack propagation, and absorption of fracture energy in the AM composite films were achieved through the synergistic effect of the hierarchical network structure, which collectively enhanced both mechanical strength and toughness.

**FIGURE 3:**
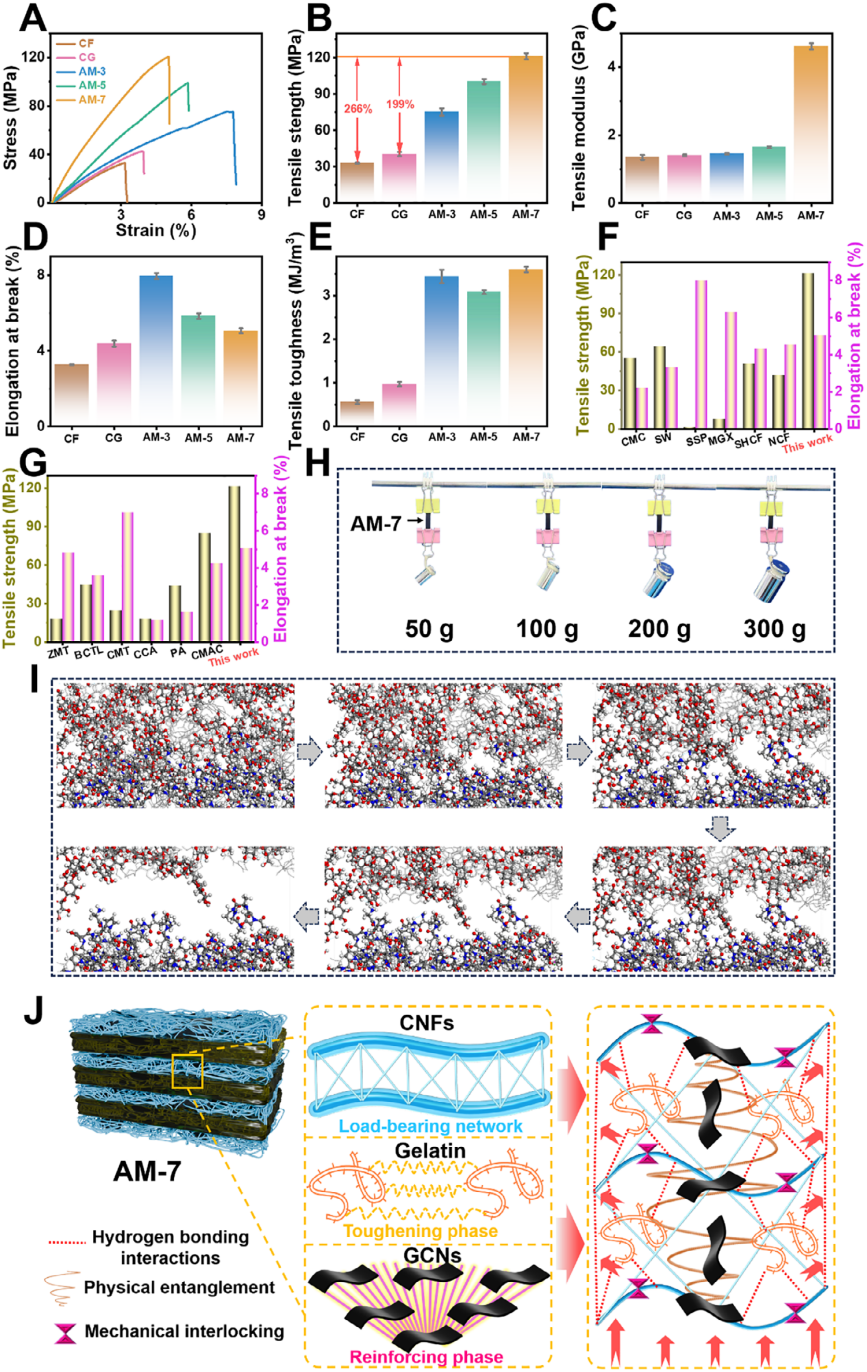
(A) Stress–strain curves, (B) tensile strength, (C) tensile modulus, (D) elongation at break, and (E) tensile toughness of CF, CG, AM‐3, AM‐5, and AM‐7 composite films. (F) Comparison of tensile strength and elongation at break of all‐biomass composites. (G) Comparison of tensile strength and elongation at break of hybrid conductive composites. (H) Schematic illustration of the tensile loading process under different applied weights (50–300 g). (I) Molecular dynamics simulation showing the deformation and interaction evolution during stretching. (J) Schematic illustration of the reinforcement mechanism of the AM composite film.

### 3.3 Thermal Management Performance

To investigate the thermal transport behavior of the AM composite films, the structural design and heat conduction pathways were systematically analyzed, as shown in Figure 4A–D. Figure 4A presents the schematic structure of the AM composite films and defines the in‐plane and out‐of‐plane thermal transport directions. As shown in Figure 4B,C, the AM composite films exhibit strong anisotropic thermal conduction behavior. The in‐plane thermal conductivities of CF, CG, AM‐3, AM‐5, and AM‐7 composite films are 0.018, 3.69, 5.62, 6.47, and 6.34 W·m^−^^1^·K^−^^1^, respectively, while the corresponding out‐of‐plane values are 0.01, 0.25, 0.33, 0.35, and 0.36 W·m^−^^1^·K^−^^1^. The in‐plane thermal conductivity increases initially and then reaches a plateau with slight fluctuations, which can be attributed to the balance between continuous network formation and interfacial scattering. The out‐of‐plane thermal conductivity shows a gradual increase with minor variations due to the competition between enhanced heat transfer pathways and interfacial thermal resistance. For further visualization of the through‐plane thermal transport behavior, finite element simulations were performed to evaluate the temperature distribution across different samples under identical heating conditions. As can be seen from Figure 4D, the bottom surface temperatures of CF, AM‐3, AM‐5, and AM‐7 composite films reach 367, 379, 389, and 395 K under a constant heat source of 400 K. The significantly higher bottom temperatures observed for the AM composite films compared with CF indicate enhanced through‐plane heat transfer. Moreover, the higher bottom temperature observed for multilayer films demonstrates improved thermal transport pathways within the multilayer structure. The interfacial thermal transport mechanism can be further explained by the structural characteristics illustrated in Figure 4E. Efficient in‐plane phonon transport was achieved due to the fact that the layered architecture constructs continuous in‐plane thermal pathways through the CG network [46]. Meanwhile, the GG interlayer functioned as an interfacial coupling region that regulated interlayer heat transfer through hydrogen bonding and physical interactions [47]. In addition, the abundant heterogeneous interfaces induced strong phonon scattering and thermal resistance, effectively suppressing out‐of‐plane heat transport and resulting in pronounced anisotropic thermal conduction behavior [48]. Overall, the thermal transport behavior is governed by the competition between continuous in‐plane pathway formation and interfacial phonon scattering, leading to a regulated and highly anisotropic heat transfer performance.

**FIGURE 4:**
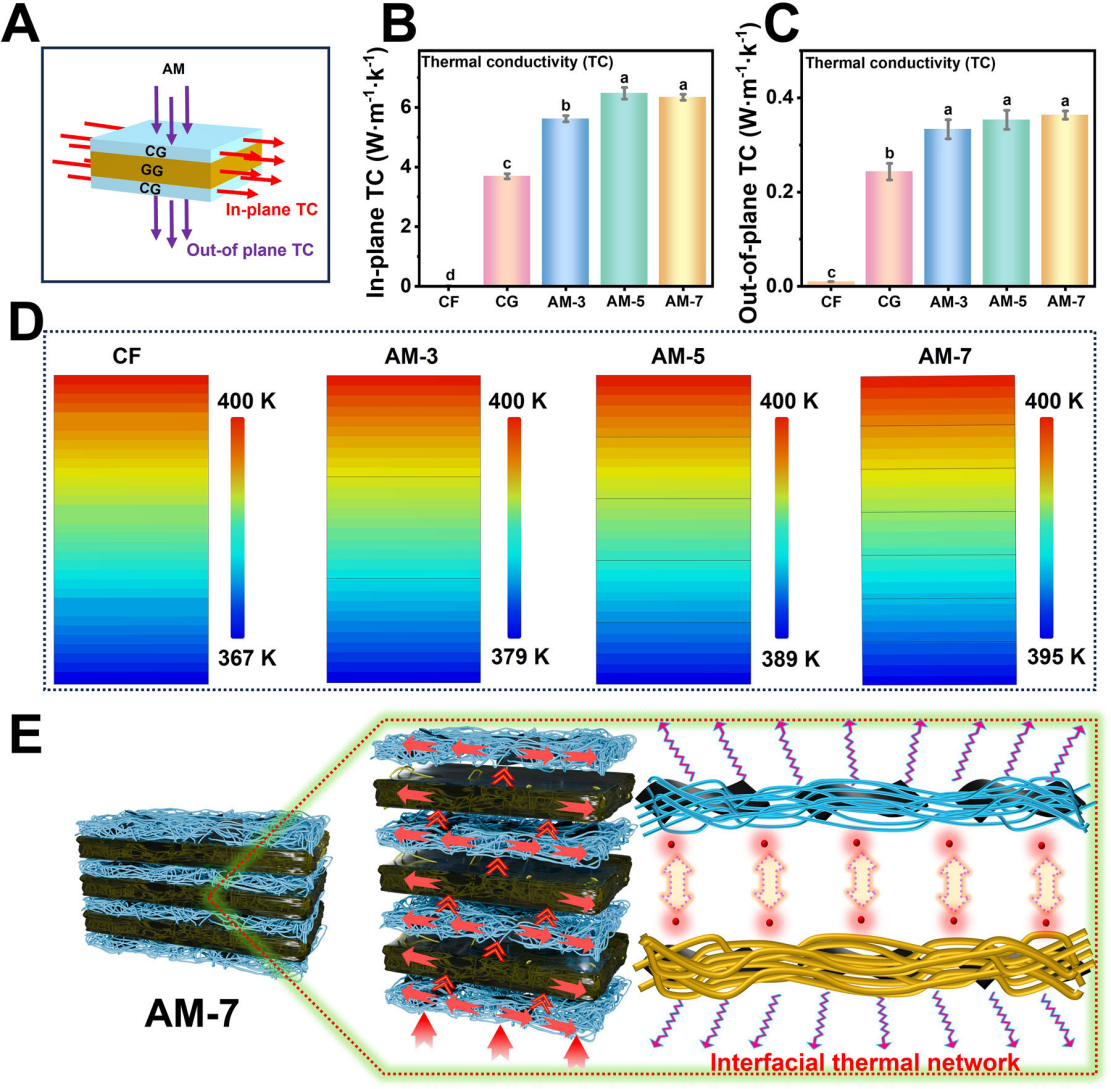
(A) Schematic illustration of anisotropic heat conduction pathways in the layered structure. (B) In‐plane thermal conductivity and (C) out‐of‐plane thermal conductivity of CF, CG, AM‐3, AM‐5, and AM‐7 composite films. (D) Finite element analysis of temperature distribution in CF, AM‐3, AM‐5, and AM‐7 composite films under identical heating conditions. (E) Schematic illustration of the interfacial thermal network and heat transfer mechanism in the AM‐7 composite film.

### 3.4 Electromagnetic Interference Shielding Performance

Figure 5A displays the frequency‐dependent EMI shielding effectiveness (SE) of CF, CG, and AM composite films in the X‐band region. Having relatively stable shielding performance across the entire frequency range suggests efficient and broadband electromagnetic attenuation behavior. As summarized in Figure 5B, the average EMI SE values of CG, AM‐3, AM‐5, and AM‐7 composite films are 35.04, 42.57, 49.94, and 56.35 dB, respectively. A significant improvement is observed in the AM composite films compared with CG, which confirms the effectiveness of the multilayer structural design in enhancing EMI shielding performance. As shown in Figure 5C, the SE_A_/SE_R_ values above 3 for CG, AM‐3, AM‐5, and AM‐7 composite films indicate that absorption loss contributes more significantly than reflection loss to the attenuation of electromagnetic energy entering the composite films. It is noteworthy that the highest SE_A_/SE_R_ value of AM‐7 composite film is attributed to the fact that the synergistic effects of the conductive network and interfacial polarization within the multilayer architecture enhance electromagnetic wave dissipation. Figure 5D reveals that the reflection coefficient (R) increases whereas the absorption coefficient (A) decreases with increasing structural complexity. This variation reflects a regulated electromagnetic energy dissipation process, in which incident electromagnetic waves are dissipated through reflection and absorption associated with the conductive network and interfacial polarization within the multilayer architecture. As shown in Figure 5E, the electromagnetic shielding efficiency of all samples exceeds 99.96%. Notably, the shielding efficiency increases progressively from CG to AM‐7, with a maximum value of 99.9998% achieved for the AM‐7 composite film, indicating near‐complete electromagnetic wave blocking. The CG composite film exhibits an electrical conductivity of 5.2 S cm^−^^1^, while the AM‐3, AM‐5, and AM‐7 composite films show values of 5.0, 4.7, and 4.1 S·cm^−^^1^, respectively (Figure S2). The slight decrease with increasing layer number may be attributed to increased interfacial resistance, while the relatively high conductivity indicates that effective conductive pathways were maintained within the multilayer films. The frequency‐dependent complex permittivity and dielectric loss tangent of the composite films are presented in Figure S3. ε′ and ε″ generally decrease with increasing frequency, while the AM composite films exhibit higher ε′, ε″, and tanδ values than the CG composite film. This behavior is mainly attributed to enhanced interfacial polarization and conductive loss arising from increased heterogeneous interfaces and interconnected GCNs networks, further supporting the contribution of dielectric loss to the EMI shielding performance. The comparison of EMI shielding effectiveness and thickness of this work with previously reported materials (Carbon foam [49], CMP/CS [50], CW/Ni [51], EP/CDW/Ag [52], MXene/wood composite [53], and WC/Co‐1000 [54]) is presented in Figure 5F. It can be observed that the AM‐7 composite film achieves a high EMI SE at a relatively low thickness, which demonstrates superior shielding efficiency compared with most reported counterparts. An electromagnetic field detector was used to measure the radiation intensity of electronic devices for further evaluating the electromagnetic shielding performance of the composite films, as shown in Figure 5G,H. The electromagnetic field detector recorded a rapid increase in electromagnetic radiation intensity upon activation of the Tesla coil, reaching peak values of 752 V m^−^^1^ and 0.72 µT, accompanied by an audible alarm signal. Upon insertion of a paper card of identical size between the Tesla coil and the detector, the measured electromagnetic field gradually decreased to 130 V m^−^^1^ and 0.17 µT, while the alarm signal remained activated. It is noteworthy that the electromagnetic radiation intensity was significantly reduced to only 17 V m^−^^1^ and 0.00 µT as the paper card was replaced by the AM composite film of the same dimensions, with complete suppression of the alarm signal. The electromagnetic field recovered to 684 V m^−^^1^ and 0.68 µT without any shielding materials present (Figure 5H). These results clearly demonstrate the superior practical EMI shielding performance of the AM composite film. The EMI shielding mechanism is schematically illustrated in Figure 5I. First, incident electromagnetic waves were reflected due to impedance mismatch at the surface upon interaction with the composite film. Second, the transmitted waves were attenuated through a synergistic mechanism due to the fact that conductive losses in the interconnected conductive network and interfacial polarization induced by abundant heterogeneous interfaces within the multilayer structure were involved [55, 56]. Additionally, the propagation path of electromagnetic waves was prolonged due to multiple scattering and internal reflections, which led to gradual energy dissipation [57]. Therefore, efficient electromagnetic interference shielding is achieved by effectively suppressing the propagation of electromagnetic waves.

**FIGURE 5:**
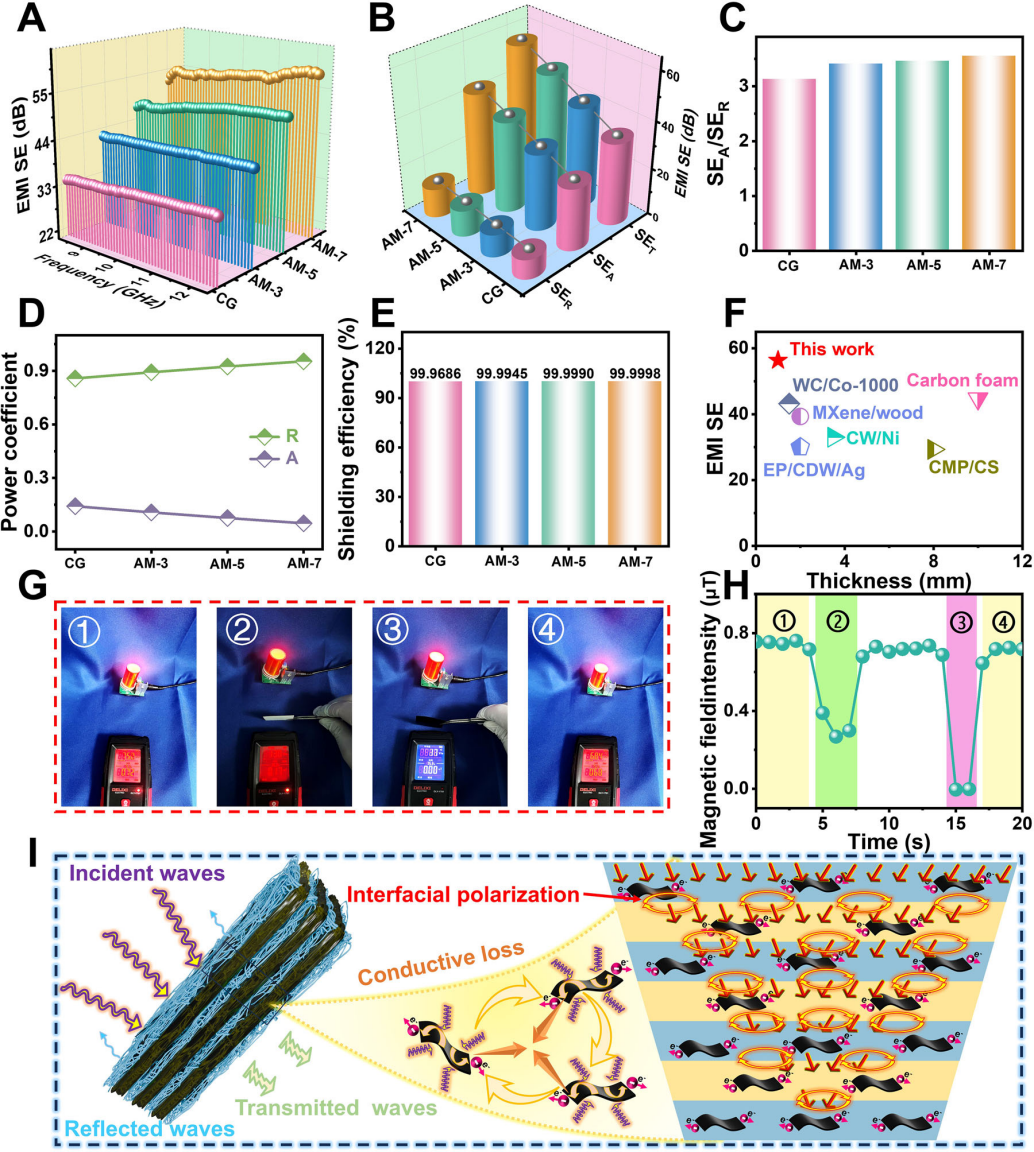
(A) EMI SE, (B) SE_A_, SE_R_, and SE_T_, (C) ratios of SE_A_/SE_R_, (D) power coefficients, and (E) EMI shielding efficiency of CG, AM‐3, AM‐5, and AM‐7 composite films in the X‐band. (F) Comparison of EMI SE and thickness with previously reported EMI shielding materials. (G) Photographic demonstration of EMI shielding performance using a portable electromagnetic field detector under different conditions. (H) Magnetic field intensity variation of AM‐7 composite film as a function of time. (I) Schematic illustration of the EMI shielding mechanism of AM composite films.

## 4 Conclusions

In this work, AM biomimetic composite films composed of CNFs, GCNs, and gelatin were successfully fabricated via a layer‐by‐layer assembly strategy inspired by the hierarchical structure of pork belly. Strong hydrogen bonding and π–π stacking interactions among the components enabled the formation of a robust hierarchical multilayer architecture, which endowed the resulting AM composite films with excellent mechanical strength, efficient thermal management capability, and outstanding EMI shielding performance. As a result, the AM‐7 composite film exhibited a tensile strength of 121.20 MPa, representing increases of 266% and 199% compared with CF and CG, respectively. In addition, a tensile modulus of 4.62 GPa, an elongation at break of 5.06%, and a tensile toughness of 3.60 MJ/m^3^ were achieved. Moreover, the composite film delivered an in‐plane thermal conductivity of 6.34 W·m^−^^1^·K^−^^1^ and an EMI shielding effectiveness of 56.35 dB demonstrating a favorable balance of mechanical robustness, thermal transport, and EMI shielding performance. This study demonstrates the great potential of high‐performance multifunctional all‐biomass based composites for next‐generation electromagnetic protection and thermal management applications.

## Author Contributions

**Junchao Ren**: conceptualization, Writing – original draft, validation, data curation. **Chenglei Huang**: conceptualization, formal analysis. **Chengxu Pei**: software, methodology. **Mengde Huang**: supervision, data curation. **Yuanyuan Xu**: methodology, formal analysis. **Chengyu Yu**: software, validation. **Hao Nie**: conceptualization, investigation. **Guangbing Zhao**: validation, visualization. **Shijing Song**: validation, data curation. **Hanwu Lei**: methodology, supervision. **Fang Wang**: conceptualization, writing – review and editing, funding acquisition. **Bin Li**: conceptualization, funding acquisition, writing – review and editing. **Qingfa Zhang**: writing – original draft, writing – review and editing, funding acquisition, conceptualization, supervision.

## Conflicts of Interest

The authors declare no conflicts of interest.

## Supporting information




**Supporting File**: advs78388‐sup‐0001‐SuppMat.docx.

## Data Availability

Data sharing not applicable to this article as no datasets were generated or analysed during the current study.
