# Supplementary material for: Dramatic Enhancement and Integration of Properties in Pork Belly‐Inspired All‐Biomass Composite Films via Facile, Sustainable, and Controllable Design
Source: Adv Sci (Weinh). 2026 Oct 22:e78388. Online ahead of print. doi: 10.1002/advs.78388 (PMC13691241; doi:10.1002/advs.78388)
Supplement: Supplementary file 1 [file ADVS-9999-e78388-s001.docx]

**Supporting Information**

**Dramatic enhancement and integration of properties in pork belly-inspired all-biomass composite films via facile, sustainable, and controllable design**

Junchao Ren^a^, Chenglei Huang^a^, Chengxu Pei^a^, Mengde Huang^a^, Yuanyuan Xu^a^, Chengyu Yu^a^, Hao Nie^a^, Guangbing Zhao^a^, Shijing Song^a^, Hanwu Lei^c^, Fang Wang^b^*, Bin Li^a^*, Qingfa Zhang^a^*

*^a^School of Mechanical and Vehicle Engineering, School of Intelligent Manufacturing, Anhui Agricultural University, Hefei 230036, China*

*^b^College of Agricultural Engineering and Food Science, Shandong University of Technology, Zibo 255049, China*

*^c^Department of Biological Systems Engineering, Washington State University, Richland 99354, United States*

∗Corresponding authors: *Email address: waterfall0711@sdut.edu.cn (F. Wang),* *libin198520@126.com (B. Li), zhangqingfa@ahau.edu.cn (Q. Zhang)*

**Table of Contents**

**Supplemental Figures**

Fig. S1. Raman spectra before and after FJH

Fig. S2. Electrical conductivity of CF, CG, AM-3, AM-5, and AM-7 composite films

Fig. S3. (A) real part of complex permittivity (ε′), (B) imaginary part of complex permittivity (ε″), and (C) dielectric loss tangent (tanδ)
